# The true effect of lithium is hard to determine

**DOI:** 10.1192/bjo.2023.572

**Published:** 2023-10-12

**Authors:** René Ernst Nielsen, Rasmus W. Licht

**Affiliations:** Department of Psychiatry, Aalborg University Hospital, Denmark; and Department of Clinical Medicine, Aalborg University, Denmark

**Keywords:** Bipolar type 1 or 2 disorders, depressive disorders, mood stabilisers, statistical methodology, complimentary therapies

## Abstract

Lithium is the primary choice for preventing bipolar disorder relapses, endorsed by guidelines. A recent systematic review by Ulrichsen et al. showed limitations in assessing its specific impact, but data supports lithium's effectiveness in managing symptoms and preventing relapse. Comprehensive guidelines and research are crucial for its continued use.

Lithium, a naturally occurring element, has stood the test of time as the gold standard for recurrence prevention in patients with bipolar disorder. Its effectiveness is so profound that most guidelines recommend it as the first-line treatment for these conditions.^1^ Beyond merely mitigating clinical mood episodes, evidence points to lithium's ability to reduce suicide and suicide-related mortality in patients undergoing treatment.^2,3^ But its value extends beyond bipolar disorder: lithium is also used in the acute and preventive treatment of patients with unipolar major depressive disorder, including those with treatment-resistant depression.^4,5^ As a maintenance treatment for bipolar disorder, lithium surpasses placebo and is comparable to other active comparators, even when those comparators were evaluated under enriched study conditions.^6^ Here, participants had initially responded to the comparator drug and were then randomised to continue that drug, switch to lithium or receive a placebo.^6^ This study design will inevitably enrich the chance of a positive outcome for participants randomised to continue a treatment for which they have responded and tolerated, and lithium's non-inferiority under these conditions is even more impressive.

Respected experts in the field of lithium treatment have been vocal advocates for its prioritisation not only as a first-line option, but also as the primary treatment offered to patients with bipolar disorder.^7^ This strong endorsement is rooted in the abundance of compelling evidence showcasing lithium's remarkable benefits in managing mood disorders. However, despite these expert opinions, the utilisation of lithium seems to have faltered, and it is crucial to delve into the potential factors contributing to this concerning trend.^8^

One major hurdle hindering lithium's broader adoption is the apprehension surrounding its side-effects. Like any medication, lithium is not without its risks, and concerns about adverse reactions have contributed to hesitancy among both patients and healthcare providers.^9^ These worries are understandable, as lithium's therapeutic index is relatively narrow, meaning that there is a fine line between an effective dose and one that may lead to toxicity, in both the short term and long term. Consequently, meticulous monitoring is essential to ensure its safe and optimal use. However, advancements in medical knowledge and technology have improved our ability to manage lithium treatment effectively, minimising the risk of adverse events.^9^

Another reason for the dwindling use of lithium lies in a lack of knowledge among healthcare practitioners regarding its proper administration and management.^10^ Lithium therapy demands specialised expertise, and not all healthcare professionals may feel adequately equipped to prescribe and monitor this treatment. Inadequate training and unfamiliarity with lithium's nuances might lead to a reluctance to incorporate it into treatment plans.

To address these issues and promote the safe and effective use of lithium, comprehensive guidelines should be developed, explicitly focusing on the management of side-effects and optimal dosing strategies. By providing clinicians with clear and up-to-date recommendations, such guidelines can bolster their confidence in utilising lithium as a therapeutic option. Additionally, enhancing medical education to encompass in-depth training on lithium therapy will empower healthcare professionals with the knowledge and skills necessary to navigate its complexities. Furthermore, raising awareness among patients about the benefits of lithium treatment, and dispelling myths surrounding its adverse effects, can foster a more informed decision-making process.^10^ Educated patients who actively participate in their treatment choices are more likely to embrace lithium as a valuable option for managing their mood disorder.

In this context, the recommendations put forth by Kovacs et al^11^ on the management of calcium and parathyroid hormone changes, by Nielsen et al^12^ concerning the renal effects of lithium, and by Tondo et al^9^ on the general management and initiation of lithium treatment, are of importance. Their insights contribute to the development of guidelines that directly tackle the challenges associated with lithium usage. These guidelines can serve as a roadmap for clinicians, offering practical strategies for mitigating side-effects and optimising treatment outcomes.

Recently, Ulrichsen et al conducted a systematic review and meta-analysis, delving into the overall clinical efficacy of lithium treatment in individuals diagnosed with bipolar disorder.^13^ Their comprehensive investigation encompassed various study designs, including randomised controlled studies, non-randomised two-arm studies and non-randomised one-arm studies, thereby involving a substantial participant cohort of 30 542 individuals. Notably, the largest proportion of participants (24 052 individuals) were included from the non-randomised two-arm studies.

The findings of Ulrichsen et al revealed interesting results, with nearly two-thirds of patients experiencing a significant response to treatment, showing a remarkable reduction of at least 50% in symptom severity from baseline during the follow-up period, irrespective of whether the episode was depressive or manic.^13^ Additionally, approximately a quarter of patients exhibited response defined as not necessitating any further medical intervention. Interestingly, when examining continuous outcomes, the most pronounced effect size was observed for mania, with a pooled Hedges’ *g*-statistic of 1.85, and for depression, with a pooled Hedges’ *g*-statistic of 1.56, surpassing the effect sizes typically observed in randomised controlled trials. The inclusion of various study designs, including both randomised and non-randomised trials, alongside one-arm studies, warrants attention to the influence of time and non-pharmacological factors on treatment outcomes. For instance, the effect of time may be greater in participants experiencing mania, where the time to the occurrence of a new similar episode might be more prolonged. Conversely, the risk of a new depressive episode might be numerically larger, which could potentially account for some of the observed outcomes in the within-group analysis.

Nevertheless, the diversity in study designs, treatment durations, outcomes and patient population, which comprised individuals exclusively diagnosed with bipolar disorder and those with co-occurring disorders, introduces conceptual heterogeneity, rendering the interpretation of results challenging and limiting their generalisability. Furthermore, Ulrichsen et al chose to present their findings solely as within-group changes, which aligns with an individual patient-focused perspective (i.e. which effect the individual patient perceives).^13^ However, this approach impedes the ability to ascertain the specific effect of the intervention, in this case, lithium. To better comprehend the impact of time on treating bipolar disorder, we shall use an analogy with the common cold. When we have a cold, various treatments such as rest, fluids and medication can make us feel better over time. If we have a long follow-up period, we might think that all treatments are effective, as all or almost all cases are cured. However, bipolar disorder is different. It is a chronic mental disorder characterised by episodes of mania and depression. These episodes can last for a significant period. Depending on duration of follow-up, we might have difficulties in interpretating results, as shorter follow-up duration (e.g. for mania) could result in overly positive results of specific treatment (e.g. lithium) when not compared with an active comparator, in a design not adjusting for the effects of time and non-pharmacological interventions like hospital admission. When we extend the follow-up time for bipolar disorder, the situation becomes more complicated. Combining the results from different study designs and duration of follow-up thereby makes generalisability harder.

In conclusion, lithium treatment is the gold standard for preventing recurrence in patients with bipolar disorder, backed by robust evidence and recommended as a primary therapy. Its potential also extends to treating unipolar major depressive disorder, even in cases of treatment-resistant depression. Nonetheless, there has been a concerning decline in lithium use. The systematic review and meta-analysis by Ulrichsen et al offers valuable insights into lithium's overall efficacy, revealing that nearly two-thirds of patients experience significant responses, with marked symptom reductions during follow-up, regardless of episode type. These results emphasise lithium's potential to substantially improve mood disorders and enhance quality of life. However, the review also underscores the necessity for cautious interpretation, because of varying study designs and patient characteristics. In addressing the challenges with declining use of lithium, comprehensive guidelines for managing side-effects and treatment strategies are pivotal. By strengthening medical education and fostering awareness among healthcare professionals and patients, we can amplify confidence in lithium's pivotal role in mood disorder therapy.
